# P38-DAPK1 axis regulated LC3-associated phagocytosis (LAP) of microglia in an in vitro subarachnoid hemorrhage model

**DOI:** 10.1186/s12964-023-01173-6

**Published:** 2023-07-21

**Authors:** Xiang-Xin Chen, Tao Tao, Xun-Zhi Liu, Wei Wu, Jin-Wei Wang, Ting-Ting Yue, Xiao-Jian Li, Yan Zhou, Sen Gao, Bin Sheng, Zheng Peng, Hua-Jie Xu, Peng-Fei Ding, Ling-Yun Wu, Ding-Ding Zhang, Yue Lu, Chun-Hua Hang, Wei Li

**Affiliations:** 1 Department of Neurosurgery, Nanjing Drum Tower Hospital, Affiliated Hospital of Medical School, Nanjing University, Nanjing, China; 2 Institute of Neurosurgery, Nanjing University, Nanjing, Jiangsu Province China; 3 Department of Neurosurgery, Nanjing Drum Tower Hospital Clinical College of Nanjing Medical University, Nanjing, Jiangsu Province China; 4 Department of Neurosurgery, Nanjing Drum Tower Hospital Clinical College of Xuzhou Medical University, Xuzhou, Jiangsu Province China; 5 Department of Neurosurgery, Nanjing Drum Tower Hospital Clinical College of Jiangsu University, Nanjing, Jiangsu Province China

**Keywords:** DAPK1, P38, LC3-associated phagocytosis, Microglia, Subarachnoid hemorrhage

## Abstract

**Background:**

The phagocytosis and homeostasis of microglia play an important role in promoting blood clearance and improving prognosis after subarachnoid hemorrhage (SAH). LC3-assocaited phagocytosis (LAP) contributes to the microglial phagocytosis and homeostasis via autophagy-related components. With RNA-seq sequencing, we found potential signal pathways and genes which were important for the LAP of microglia.

**Methods:**

We used an in vitro model of oxyhemoglobin exposure as SAH model in the study. RNA-seq sequencing was performed to seek critical signal pathways and genes in regulating LAP. Bioparticles were used to access the phagocytic ability of microglia. Western blot (WB), immunoprecipitation, quantitative polymerase chain reaction (qPCR) and immunofluorescence were performed to detect the expression change of LAP-related components and investigate the potential mechanisms.

**Results:**

In vitro SAH model, there were increased inflammation and decreased phagocytosis in microglia. At the same time, we found that the LAP of microglia was inhibited in all stages. RNA-seq sequencing revealed the importance of P38 MAPK signal pathway and DAPK1 in regulating microglial LAP. P38 was found to regulate the expression of DAPK1, and P38-DAPK1 axis was identified to regulate the LAP and homeostasis of microglia after SAH. Finally, we found that P38-DAPK1 axis regulated expression of BECN1, which indicated the potential mechanism of P38-DAPK1 axis regulating microglial LAP.

**Conclusion:**

P38-DAPK1 axis regulated the LAP of microglia via BECN1, affecting the phagocytosis and homeostasis of microglia in vitro SAH model.

Video Abstract

**Supplementary Information:**

The online version contains supplementary material available at 10.1186/s12964-023-01173-6.

## Introduction

As a form of hemorrhagic stroke, subarachnoid hemorrhage (SAH) is known as the high mortality, the high morbidity and the high economy burden on society [1, 2]. The main etiology of SAH is rupture of aneurysms and the characteristic is that the blood broken into subarachnoid space stimulates cerebral cortex continuously causing a series of pathological changes [2]. Some studies have revealed the important relationship between residual blood in subarachnoid space and prognosis of SAH patients [3, 4]. Therefore, blood clearance is becoming a focus of research on SAH, and microglial phagocytosis plays an important role in promoting endogenous absorption of blood [5–8].

Previous studies on microglia phagocytosis have mainly focused on three pathways including “find-me”, “eat-me” and “don’t-eat-me” that are chemotactic signals, ligand-receptor-activated phagocytic signals and phagocytic inhibitory signals [9]. These studies took microglia as a tool to remove metabolic wastes and toxins from the nervous system. Few studies have examined how microglia return to self-balance after phagocytosis although it is important for the central nervous system to have homeostatic microglia in the disease state [10]. Lysosomes as the primary degradative compartment within cells are dynamic regulator of cell and organismal homeostasis, and the fusion of phagosomes and lysosomes is necessary for microglial homeostasis [11]. Therefore, how to promote the fusion of phagosomes and lysosomes in microglia after SAH is what we mainly want to study which we call “digest me”.

LAP is a process of autophagy-related proteins induced fusion of phagosomes and lysosomes, which involves maturation of phagosomes and degradation of phagocytic contents [12–15]. Researches have shown that LAP regulated phagocytosis and inflammation of myeloid cells [16, 17]. In addition, LAP was identified to regulate the homeostasis of astrocyte and retinal pigment epithelium [18, 19]. Therefore, we think that LAP is a promising biological process in regulating microglial phagocytosis and homeostasis. However, there are few studies on the LAP of microglia, and there is no effective molecule in regulating the microglial LAP. LAP is dominated by autophagy-related proteins, and inhibited autophagy impaired the LAP of microglia [13, 20]. In our study, we found that the phagocytosis and autophagy of microglia were inhibited in vitro SAH model. Therefore, we want to regulate the microglial LAP based on autophagy to improve the phagocytosis and homeostasis of microglia after SAH.

With RNA-seq sequencing, we found the significantly changed signal pathways and genes associated with autophagy in vitro SAH model including mitogen-activated protein kinase (MAPK) signaling pathway and death associated protein kinase 1 (DAPK1). The MAPK signal pathway including P38 pathway, ERK pathway and JNK pathway has been confirmed to be associated with autophagy [21–23]. DAPK1, as a stress-responsive serine/threonine (Ser/Thr) kinase, plays an important role in cell death and autophagy [24]. Oikonomou et al. (2016) indicated that DAPK1 contributed to LAP, but potential mechanisms were not explored [25]. In the article, we found that P38-DAPK1 axis was related to the phagocytosis and homeostasis of microglia and regulated the LAP of microglia via BECN1 in vitro SAH model.

## Methods

### Cell culture

To get primary microglia, the cortex from newborn mice within 24 h was harvested and digested. The animal procedures were approved by the Ethics Review Committee for Animal Experimentation at Nanjing Drum Tower Hospital. Microscope was used to remove meninges and TrypLE was used to digest the cortex for 10 min in a 37℃ incubator. Dulbecco's Modified Eagle Medium (DMEM, C11995500BT, Gibco, CA, USA) containing 10% Fetal Bovine Serum (FBS, 10,099,141, Gibco, CA, USA) and 1% penicillin–streptomycin (10,378,016, Gibco, CA, USA) was used to culture primary microglia which were available at 10 days in vitro (DIV10) and DIV13. Microglia were floating after the plates shaken gently and were transferred to new plates. BV2 (ZQ0397, Zhong Qiao Xin Zhou Biotechnology, Shanghai, China) as microglial clone were cultured in DMEM containing 10% FBS and 1% penicillin–streptomycin and cultured every other day.

### Oxyhemoglobin-induced SAH model in vitro

To mimic SAH in vitro, the microglia and BV2 cells were exposed to oxyhemoglobin (oxyHb, 50,200, Kamai Shu Biotechnology, Shanghai, China) at a concentration of 10 μmol/L as previously described [26, 27].

### RNA interference

BV2 cells were transfected with plasmid expressing *Dapk1*-specific shRNA. Pre-experiment revealed the optimal concentration of plasmid and lipofectamine (11,668,019, Thermo Fisher Scientific, MA, USA). After cells transfected for 4 h, cell medium was replaced with complete medium. 2 days later, cells were performed in further research. The *Dapk1* shRNA and negative control sequences were as follows: *Dapk1-i1* sequence, 5’-GGAGGCAACGGAATTCCTTAA-3’; *Dapk1-i2* sequence, 5’-GCCTAAAGACACCCAACAAGC -3’; *Dapk1-i3* sequence, 5’-GCATGGGACACCTCCATTACT -3’; *NC* sequence, 5’- TTCTCCGAACGTGTCACG T -3’. BLAST research identified that the above-mentioned sequences had no important homology with other mouse genes.

### Western blot analysis

RIPA lysis buffer (89,901, Thermo Scientific, MA, USA) supplemented with protease inhibitor (GRF101, Epizyme, Shanghai, China) and phosphatase inhibitor (GRF102, Epizyme, Shanghai, China) was added to lyse microglia. BCA Protein Assay Kit (P0012S, Beyotime, Nanjing, China) was used to quantitatively calculate protein concentrations. PAGE Gel Fast Preparation Kit (PG111 & PG113, Epizyme, Shanghai, China) was used to separate proteins and polyvinylidene difluoride membrane (3,010,040,001, Sigma, MO, USA) was used to transfer the proteins. The membrane was blocked in 5% skim milk for 2 h at room temperature and was incubated overnight at 4℃ with primary antibodies. Thereafter, the membrane was washed in TBST and incubated with horse-radish peroxidase (HRP)-conjugated secondary antibodies for 2 h at room temperature. The developing solution (WBKLS0500, Sigma, MO, USA) was used to detect protein bands and Fiji software (National Institutes of Health, Bethesda, MD, USA) was used to analyze the bands. Antibodies used are listed in Table 1.

**Table 1:** Antibodies used in the study

Protein	Product code	Application	Dilution ratio	Company	Affiliating area
DAPK1	25136–1-AP	WB	1:2000	Proteintech	Wuhan, China
DAPK1	67815–1-Ig	IF	1:200	Proteintech	Wuhan, China
BECN1	11306–1-AP	WB IF	1:2000 1:200	Proteintech	Wuhan, China
RUBICON	21444–1-AP	WB IF	1:2000 1:200	Proteintech	Wuhan, China
p-ERK	4370	WB	1:1000	Cell Signaling Technology	MA, USA
ERK	4695	WB	1:1000	Cell Signaling Technology	MA, USA
p-JNK	9255	WB	1:1000	Cell Signaling Technology	MA, USA
JNK	9252	WB	1:1000	Cell Signaling Technology	MA, USA
p-P38	4511	WB	1:1000	Cell Signaling Technology	MA, USA
P38	8690	WB	1:1000	Cell Signaling Technology	MA, USA
ATG5	9980	WB	1:1000	Cell Signaling Technology	MA, USA
ATG7	8558	WB	1:1000	Cell Signaling Technology	MA, USA
LC3A/B	4108S	WB	1:2000	Cell Signaling Technology	MA, USA
P62	23214S	WB	1:2000	Cell Signaling Technology	MA, USA
β-ACTIN	4970	WB	1:5000	Cell Signaling Technology	MA, USA
Anti-rabbit IgG, HRP-linked Antibody	7074S	WB	1:5000	Cell Signaling Technology	MA, USA
Anti-mouse IgG, HRP-linked Antibody	7076S	WB	1:5000	Cell Signaling Technology	MA, USA
Mouse Anti-Rabbit IgG (Light-Chain Specific)	93702S	WB	1:2000	Cell Signaling Technology	MA, USA
LAMP1	1D4B	WB	1:1000	Developmental Studies Hybridoma Bank	IA, USA
Anti-Ubiquitin antibody	ab7780	WB	1:1000	abcam	London, UK

### Immunoprecipitation analysis

Cells were lysed with cell lysis buff containing Tris–HCl (RES3098T-B7, Sigma, MO, USA), NaCl (204,439, Sigma, MO, USA), EDTA (03609, Sigma, MO, USA), Triton X-100 (X100, Sigma, MO, USA), Glycerol (G5516, Sigma, MO, USA), double distilled water and protease inhibitor (GRF101, Epizyme, Shanghai, China). Protein A/G plus-agarose beads (sc-2003, Santa Cruz, SC, USA) were used to preclean the cell lysates at 4℃ for 0.5 h. Thereafter the supernatant was incubated with the specific antibodies against BECN1 at 4℃ overnight. Protein A/G agarose beads were used to incubate the suspension at 4℃ for 2 h on the second day. Immunocomplexes were washed with the cell lysis buff, followed by WB. Antibodies used are listed in Table 1.

### Quantitative real-time polymerase chain reaction

Total RNA Extraction Reagent (R401-01, Vazyme, Nanjing, China) was used to extract total mRNA, and reverse transcription mix (R223-01, Vazyme, Nanjing, China) was used to reverse mRNA into cDNA. SYBR Green Master Mix (Q331, Vazyme, Nanjing, China) was used to perform qPCR. The results were analyzed with 2^*−△△Ct*^ method and the quantity of *Gapdh* was used for normalization. Primers used are listed in Table 2.

**Table 2:** Primers used in the study

Gene	Forward primer	Reverse primer
*Il-1b*	GAAATGCCACCTTTTGACAGTG	TGGATGCTCTCATCAGGACAG
*Il-6*	TAGTCCTTCCTACCCCAATTTCC	TTGGTCCTTAGCCACTCCTTC
*Nlrp3*	ATTACCCGCCCGAGAAAGG	TCGCAGCAAAGATCCACACAG
*Dapk1*	ATGACTGTGTTCAGGCAGGAA	CCGGTACTTTTCTCACGACATTT
*Dapk2*	AGGCGTCATCACCTACATCC	GAGCCTCTTGGATTGTGAGC
*Dapk3*	ATGTCCACATTCAGGCAAGAG	CCTCGCGTTCGATCTCCTC
*Becn1*	ATGGAGGGGTCTAAGGCGTC	TCCTCTCCTGAGTTAGCCTCT
*Rubicon*	GATGGGGAGCGTCTGCTAGA	AGTCGTCTTCAAATTACCCAGC
*Atg5*	TGTGCTTCGAGATGTGTGGTT	GTCAAATAGCTGACTCTTGGCAA
*Atg7*	GTTCGCCCCCTTTAATAGTGC	TGAACTCCAACGTCAAGCGG
*Ctsb*	TCCTTGATCCTTCTTTCTTGCC	ACAGTGCCACACAGCTTCTTC
*Ctsl*	ATCAAACCTTTAGTGCAGAGTGG	CTGTATTCCCCGTTGTGTAGC
*Gapdh*	AGGTCGGTGTGAACGGATTTG	TGTAGACCATGTAGTTGAGGTCA
*Dait4*	CAAGGCAAGAGCTGCCATAG	CCGGTACTTAGCGTCAGGG
*Prkcq*	TATCCAACTTTGACTGTGGGACC	CCCTTCCCTTGTTAATGTGGG

### Immunofluorescent staining

Microglia were fixed with 4% paraformaldehyde (P0099, Beyotime, Nanjing, China) and permeabilized with 0.1% Triton X-100 (X100, Sigma, MO, USA). Immunology Staining Blocking Buffer (P0102, Beyotime, Nanjing, China) was used to block non-specific antigen. The cells were incubated overnight with primary antibodies and thereafter incubated with secondary antibodies coupled with fluorescent moieties. 4,6-diamidino-2-phenylindole (DAPI, 62,248, Thermo Fisher Scientific, MA, USA) as nucleic acid dye was used to incubate cells for 10 min. pHrodo™ Green E. coli BioParticles (1:1000, P35381, Thermo Fisher Scientific, MA, USA) was used to incubate cells for 30 min. Confocal microscope (FV3000, Olympus Corporation, Tokyo, Japan) was used to take pictures and Fiji Software (National Institutes of Health, Bethesda, MD, USA) was used to analyze the pictures. The colocalization coefficients of the two proteins including Pearson’s Coefficient, Overlap Coefficient and Manders' Coefficient were analyzed by the JACoP plugin in Fiji software. Pearson’s Coefficients and Overlap Coefficient are suitable for colocalization analysis when fluorescence intensities of two proteins had a single linear relationship. Manders' Coefficient can show the overlap ratio of two proteins and is not limited to the linear relationship [28]. Antibodies used are listed in Table 1.

### Flow cytometry

After incubated in DMEM dissolved with pHrodo™ Green E. coli BioParticles (1:100, P35381, Thermo Fisher Scientific, MA, USA) for 30 min, cells were digested with trypsin and washed with PBS twice. Flow cytometer (BD Accuri™ C6 Plus, NJ, USA) was used to analyze expression of E. coli particles.

### RNA-seq sequencing

Total RNA of microglia was collected after oxyHb exposure. The expression levels of genes were calculated by the number of clean reads mapped to genomic regions using Illumina next-generation sequencing technology. The software RSEM was used to quantitatively analyze the expression levels of genes and transcripts and analyze the differential expression of genes/transcripts among different samples, which reveals the regulatory mechanism of genes by combining sequence function information. The raw data for this sequencing have been uploaded to SRA database (SRP406792).

### Statistical analysis

All data were expressed as the mean ± SEM. Prism 9.0 (GraphPad Software, La Jolla, CA, USA) was used conduct statistical analysis. Student’s t-test was used to compare the differences between two groups and one-way ANOVA was used for comparisons of more than two groups. *P* < 0.05 was considered statistically significant. Schematic diagram of our study was done in BioRender (BioRender, GTA, CAN).

## Results

### OxyHb exposure induced the excessive inflammation and impaired phagocytosis in microglia

**Fig. 1:**
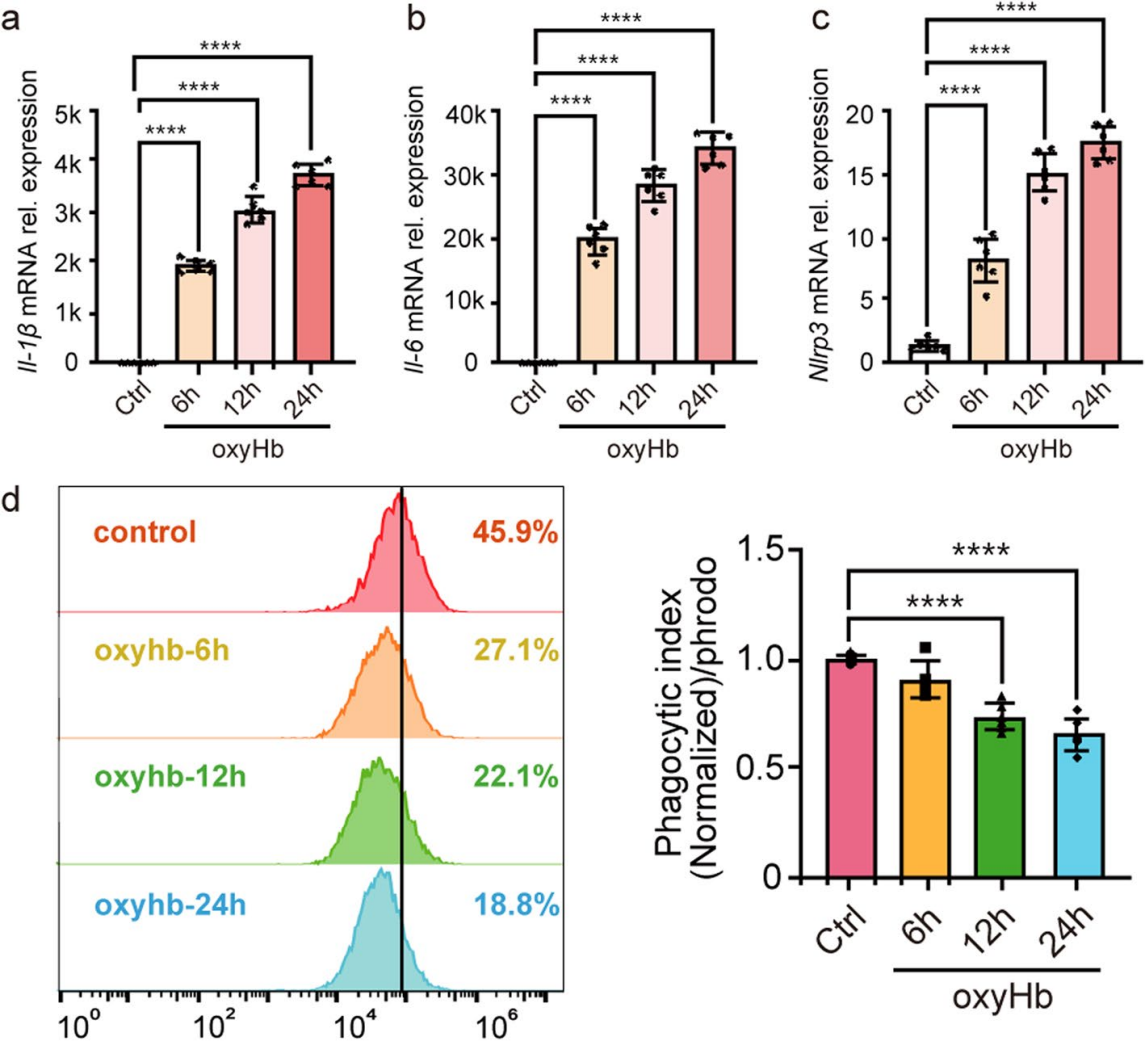
OxyHb exposure induced the excessive inflammation and impaired phagocytosis in microglia. The relative mRNA expression of *Il-1β* (**a**), *Il-6* (**b**) and *Nlrp3* (**c**) at 6 h, 12 h and 24 h after oxyHb exposure. The change of microglial phagocytosis at 6 h, 12 h and 24 h after oxyHb exposure (**d**). Data are presented as mean ± SEM. ****-*P* < 0.0001

Firstly, we need to clarify the changes of inflammation and phagocytosis in microglia after oxyHb exposure. As described previously [29], microglia were stimulated with oxyHb respectively for 6 h, 12 h and 24 h. The mRNA expression of *Il-1β*, *Il-6* and *Nlrp3* of microglia at different time points increased significantly as shown in Fig. 1a, b, c. With pHrodo™ Green E. coli BioParticles, we assessed microglial phagocytic ability in the in vitro SAH model. Accompanied by oxyHb exposure, the phagocytic ability of microglia decreased significantly (Fig. 1d).

### OxyHb exposure inhibited the LAP of microglia

**Fig. 2:**
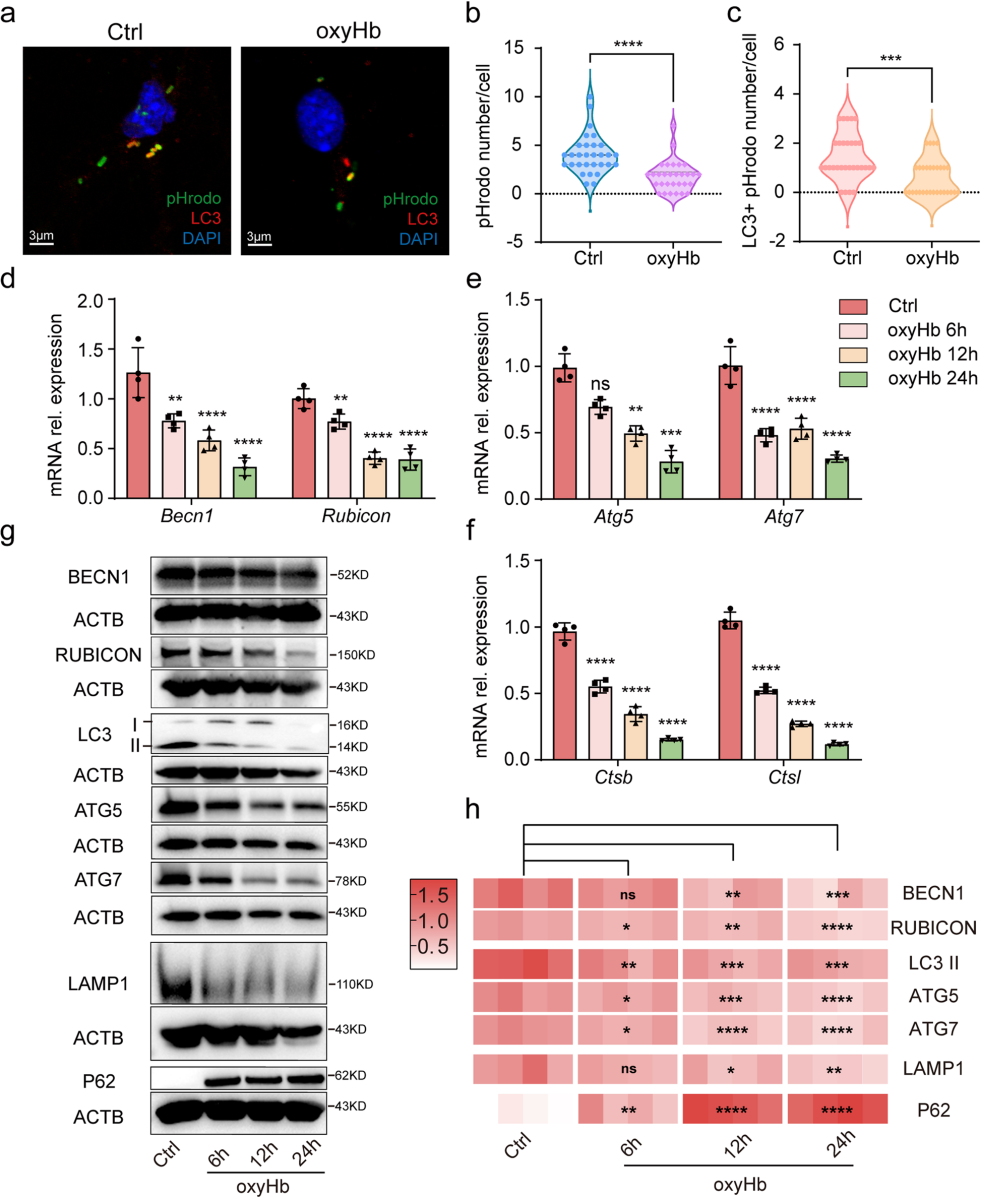
Inhibited LAP after oxyHb exposure. Immunofluorescence analysis of pHrodo and LC3 (**a**) showing the number of particles (**b**) and LC3 + particles (**c**) after oxyHb exposure. The relative mRNA expression of *Becn1* and *Rubicon* (**d**). The relative mRNA expression of *Atg5* and *Atg7* (**e**). The relative mRNA expression of *Ctsb* and *Ctsl* (**f**). Protein bands (**g**) and semi-quantitative analysis (**h**) showing the protein expression of BECN1, RUBICON, LC3 II, ATG5, ATG7, LAMP1 and P62. Data are presented as mean ± SEM. ****-*P* < 0.0001, ***-*P* < 0.001, **-*P* < 0.01, *-*P* < 0.05; ns, non-significant. Scale bar: 3 μm

Considering the association between LAP and phagocytosis, the change of microglial LAP was detected. With immunofluorescent staining (IF), the microglial phagocytosis was detected again, and the microglia in the oxyHb group phagocytosed fewer particles (Fig. 2a, b). LC3 was a specific marker of autophagosome formation and the number of LC3 encapsulated particles per cell decreased (Fig. 2a, c), which indicated that the LAP of microglia was inhibited after oxyHb exposure. Thereafter, we detected LAP-related genes and proteins in microglia after oxyHb exposure. BECN1 and RUBICON provided conditions for LC3 conjugation with phagosome and played an important role in the first stage of LAP. As shown in Fig. 2d, the mRNA expression of *Becn1* and *Rubicon* decreased significantly. With WB analysis, the protein expression of BECN1 and RUBICON were detected which was consistent with the change of genes (Fig. 2g, h). In the second stage of LAP, LC3 II formation was crucial and was associated with ATG5 and ATG7. Therefore, we detected the mRNA expression of *Atg5* and *Atg7* and found oxyHb exposure decreased the expression (Fig. 2e). The relative protein expression of LC3 II, ATG5 and ATG7 also decreased significantly (Fig. 2g, h). LAP was LC3-regulated fusion of phagosomes and lysosomes, and the function of lysosomes was important for degradation of phagocytic contents. Therefore, the last stage of LAP was accomplished in lysosome. As shown in Fig. 2f, the mRNA expression of lysosome-associated genes *Ctsb* and *Ctsl* decreased significantly. There was also significant difference of the lysosome-associated protein LAMP1 between control (Ctrl) group and oxyHb group (Fig. 2g, h). In addition, the relative protein expression of P62 increased in microglia after oxyHb exposure, which indicated the reduced autophagic flow. In conclusion, the LAP of microglia in the in vitro SAH model was inhibited in all stages.

### RNA-seq sequencing revealed significant autophagy-related signal pathways and genes

**Fig. 3:**
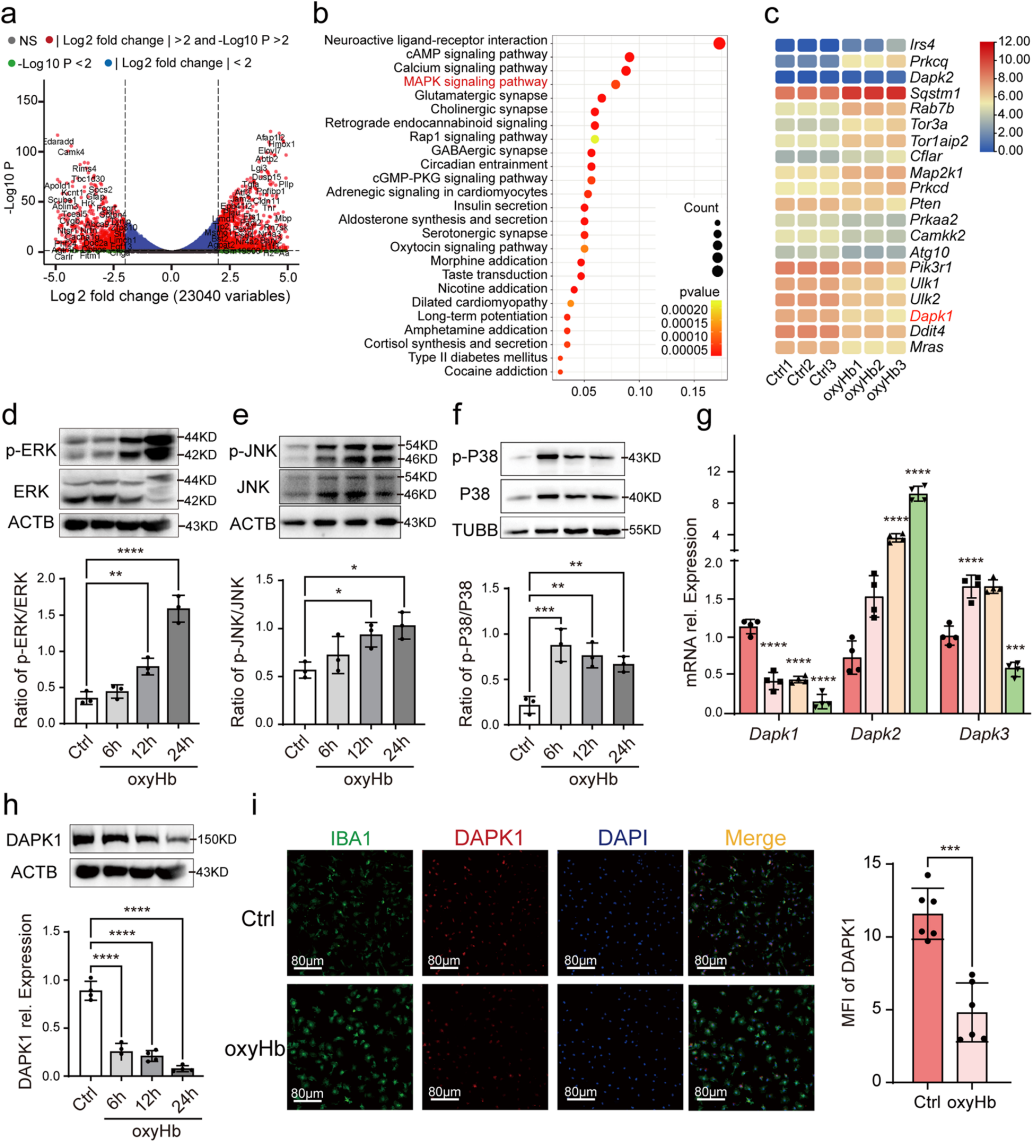
The significantly changed signal pathways and genes after oxyHb exposure. Volcano Plot showing the change of genes (**a**). KEGG pathway enrichment analysis showing the significantly changed signal pathways (**b**). KEGG-derived autophagy pathways analysis showing the significantly changed genes (**c**). The protein expression of P-ERK, ERK, P-JNK, JNK, p-P38, P38 after oxyHb exposure (**d**, **e**, **f**). The mRNA expression of *Dapk1*, *Dapk2* and *Dapk3* after oxyHb exposure (**g**). The protein expression of DAPK1 after oxyHb exposure (**h**). IF analysis showing the location and the expression of DAPK1 after oxyHb exposure (**i**). ****-*P* < 0.0001, ***-*P* < 0.001, **-*P* < 0.01, *-*P* < 0.05. Scale bar: 80 μm

With RNA-seq sequencing, we seek the potential signal pathways and genes to regulate the LAP of microglia. As shown in Fig. 3a, oxyHb-treated microglia exhibited distinct gene expression different from untreated microglia. KEGG pathway enrichment analysis revealed that the most significantly changed signal pathway associated with autophagy was MAPK pathway which included ERK pathway, JNK pathway and P38 pathway (Fig. 3b). Therefore, we detected the expression of p-ERK, ERK, p-P38, P38, p-JNK and JNK. As shown in Fig. 3d, e and f, the ratio of p-P38/P38, p-ERK/ERK, p-JNK/JNK all increased, which indicated the importance of MAPK signal pathway in microglial autophagy. Genes from KEGG-derived autophagy pathways (map04140) were extracted. We selected the top 10 genes with increased expression and decreased expression respectively based on transcript per million (TPM) of genes. As shown in Fig. 3c, we found that *Dapk* gene family including *Dapk1* and *Dapk2* changed significantly, and detected mRNA expression of *Dapk1*, *Dapk2* and *Dapk3* with qPCR. We confirmed that *Dapk1* expression decreased gradually and *Dapk2* expression increased gradually (Fig. 3g) after oxyHb exposure consistent with the results of RNA-seq sequencing. However, the expression abundance of *Dapk2* was well below that of *Dapk1* and we thought *Dapk1* played a more important role in microglial autophagy after SAH (Fig. 3c). Detailed results were shown in the Additional file 1: Table S1. With WB analysis, we detected the protein expression of DAPK1 which was consistent with the result of qPCR (Fig. 3h). In addition, IF analysis confirmed the location and expression of DAPK1 in microglia in vitro SAH model again (Fig. 3i). In fact, we also detected mRNA expression of *Ddit4* and *Mras* with qPCR which changes mildly (Additional file 2: Fig.S1). *Irs4* and *Prkcq* were excluded because of low expression. In conclusion, with RNA-seq sequencing, we found that MAPK pathway and DAPK1 had the potential of regulating autophagy and LAP of microglia in the in vitro SAH model.

### P38 inhibitor improved LAP significantly

**Fig. 4:**
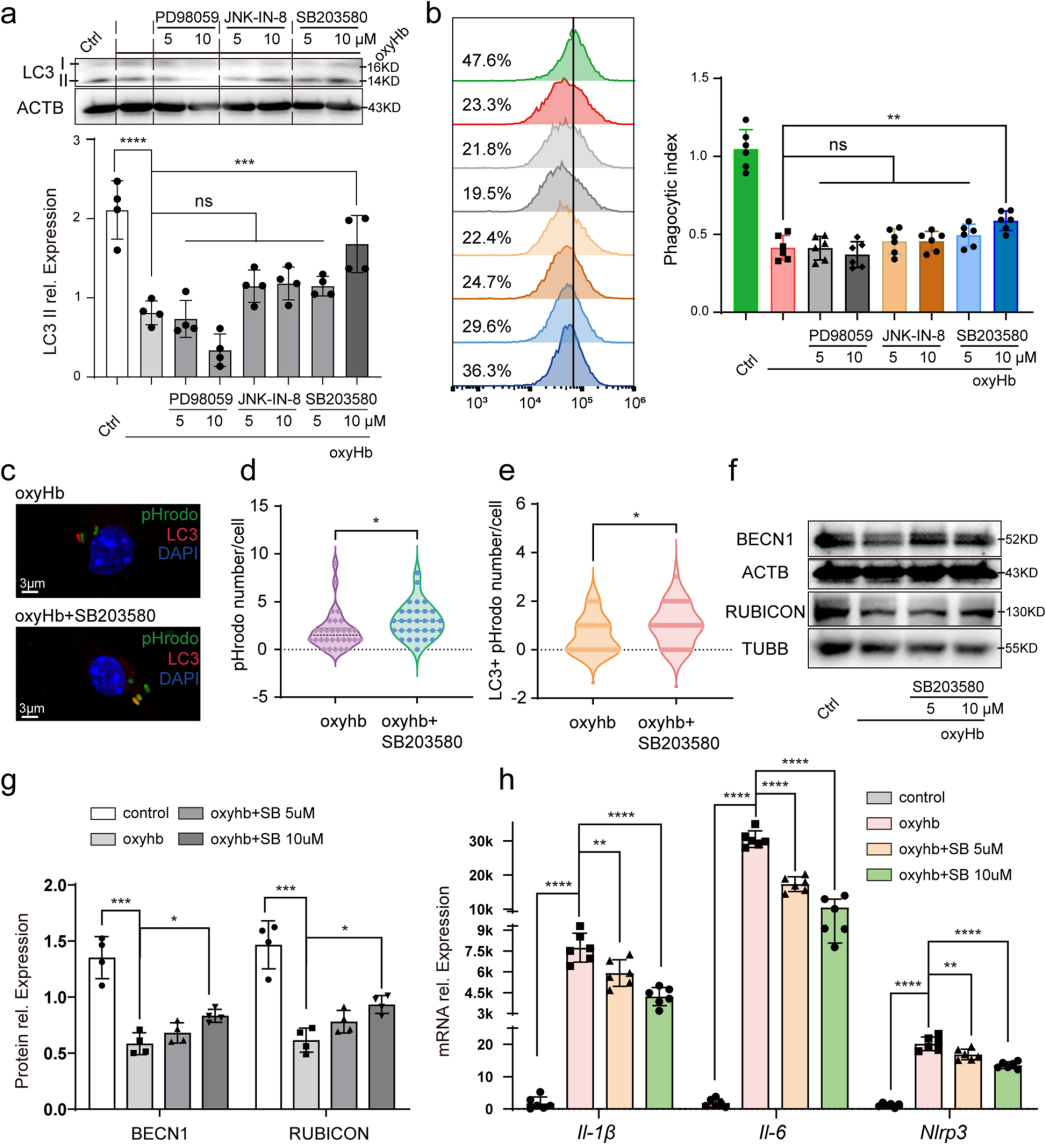
Improved LAP induced by P38 inhibitor. The protein expression of LC3 II after PD98059, JNK-IN-8 and SB203580 added (**a**). The change of microglial phagocytosis after PD98059, JNK-IN-8 and SB203580 added (**b**). Immunofluorescence analysis of pHrodo and LC3 (**c**) showing the number of particles (**d**) and LC3 + particles (**e**) after SB203580 added. Protein bands (**f**) and semi-quantitative analysis (**g**) showing the protein expression of BECN1 and RUBICON after SB203580 (SB) added. The mRNA expression of *Il-1β*, *Il-6* and *Nlrp3* after SB203580 (SB) added (**h**). ****-*P* < 0.0001, ***-*P* < 0.001, **-*P* < 0.01, *-*P* < 0.05; ns, non-significant. Scale bar: 3 μm

After finding the potential signal pathway, we need to identify the role of MAPK pathway in regulating the LAP of microglia. The inhibitors of ERK, JNK and P38 were applied in vitro SAH model to confirm the effect of MAPK signal pathway on the LAP of microglia. As shown in Fig. 4a, SB203580 as P38 inhibitor significantly increased the relative protein expression of LC3 II. However, JNK-IN-8 as JNK inhibitor and PD98059 as ERK inhibitor had no important effect on the expression of LC3 II. We also detected the change of microglial phagocytosis induced by MAPK inhibitors. SB203580 promoted microglia to phagocytosed more E.coli particles, but PD98059 and JNK-IN-8 failed to improve the phagocytosis of microglia (Fig. 4b). With IF analysis, SB203580 was confirmed again to improve phagocytic ability (Fig. 4c, d) and increased the number of LC3 encapsulated particles per cell (Fig. 4c, e), which indicated P38 contributed to LAP in microglia after SAH. In addition, to further clarify the regulating effect of P38 on LAP, we applied SB202190 as another P38 inhibitor targeting p38α/β and confirmed that SB202190 promoted the LAP of microglia (Additional file 3: Fig. S2). The most remarkable difference of LAP from canonical autophagy was the complex coupled to the phagosomes and the complex was mainly composed of BECN1 and its cofactor RUBICON [30]. Therefore, we detected the protein expression of BECN1, RUBICON with WB and found increased expression of BECN1 and RUBICON induced by SB203580 (Fig. 4f, g). In addition, the mRNA expression of *Il-1β*, *Il-6* and *Nlrp3* in oxyHb + SB203580 group decreased compared with that in oxyHb group, which revealed P38 inhibitor alleviated microglia-mediated neuroinflammation (Fig. 4h).

### P38 regulated LAP via DAPK1

**Fig. 5:**
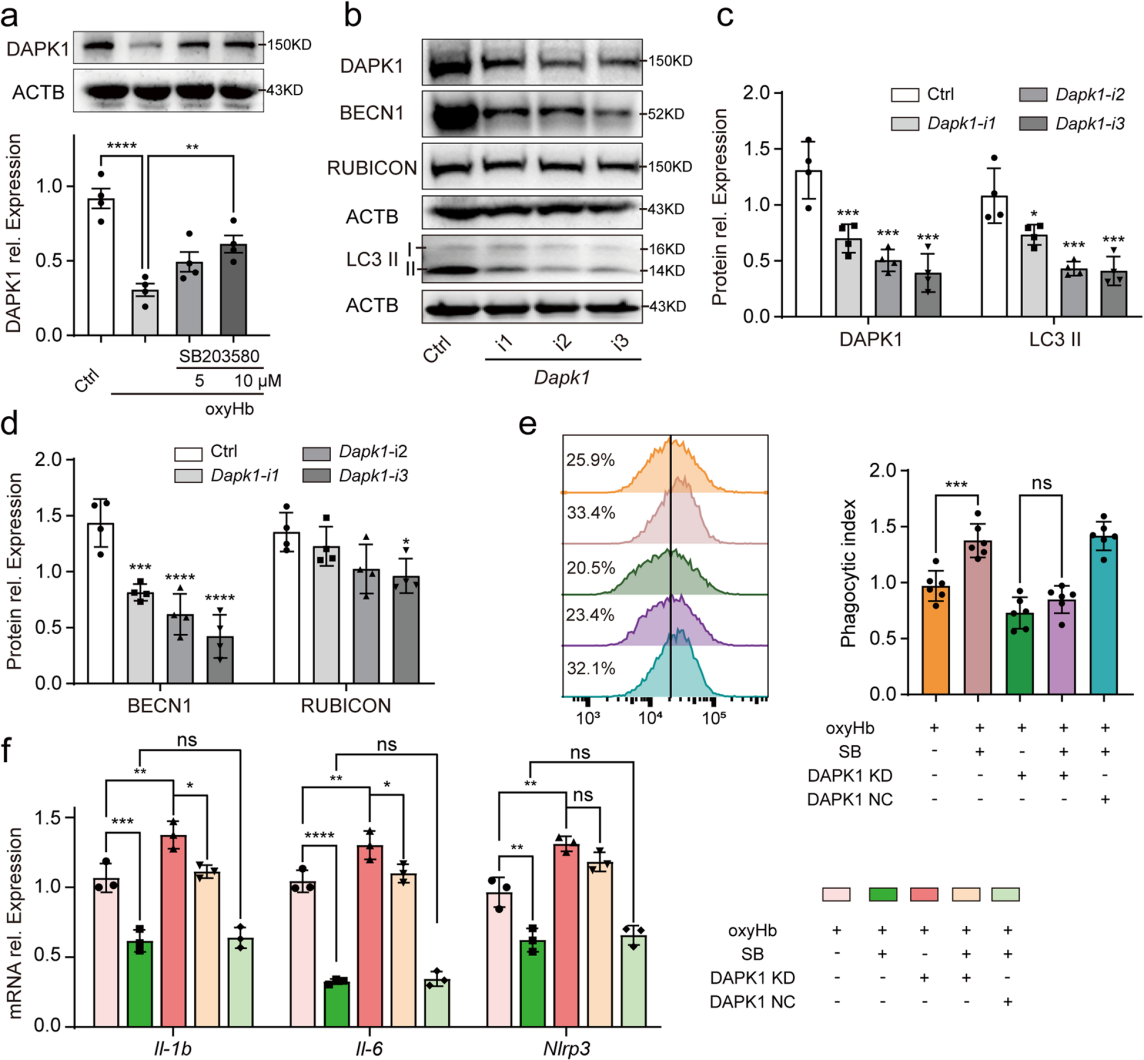
P38-regulated LAP via DAPK1. The protein expression of DAPK1 after SB203580 added (**a**). Protein bands (**b**) and semi-quantitative analysis showing the protein expression of DAPK1 (**c**), LC3 II (**c**), BECN1 (**d**) and RUBICON (**d**) after transfection of DAPK1-specific plasmid. The change of microglial phagocytosis after DAPK1 knock-down (**e**). The mRNA expression of *Il-1β*, *Il-6* and *Nlrp3* after DAPK1 knock-down (**f**). ****-*P* < 0.0001, ***-*P* < 0.001, **-*P* < 0.01, *-*P* < 0.05; ns, non-significant

With RNA-seq sequencing, we found that DAPK1 had an important potential role in microglial autophagy after SAH and we wanted to further investigate the role of DAPK1 in the LAP of microglia. Previous research indicated close association between P38 and DAPK1 [31]. Oikonomou et al. (2016) identified a potential link between DAPK1 and LAP in noncanonical fungal autophagy [25]. Therefore, we detected the expression change of DAPK1 induced by SB203580 in vitro SAH model. As shown in Fig. 5a, SB203580 increased the relative protein expression of DAPK1. Furthermore, DAPK1 expression was assessed after plasmid transfection expressing DAPK1-specific shRNA in microglia clone BV2. As shown in Fig. 5b and c, we successfully knocked down the expression of DAPK1 in which the knock-down efficiency of *Dapk1-i3* plasmid was the highest in three different shRNA plasmids. Therefore, we used *Dapk1-i3* plasmid to identify the contribution of DAPK1 in P38-regulated LAP. As shown in Fig. 5e, after transfection of *Dapk1-i3* plasmid in BV2, SB203580 failed to increased E. coli particles phagocytosed by BV2. In addition, compared with oxyHb group, BV2 in oxyHb + DAPK1 KD group phagocytosed fewer particles. We also assessed the protein expression of BECN1, RUBICON and LC3 II after transfection of three different plasmids. Interestingly, the relative protein expression of BECN1 and LC3 II decreased significantly (Fig. 5b, c, d), but the expression of RUBICON decreased slightly only after transfection of *Dapk1-i3* plasmid (Fig. 5b, c, d). Therefore, DAPK1 played a crucial role in P38-regulated LAP in microglia. Furthermore, we studied the effect of DAPK1 on P38-regulated neuroinflammation. As shown in Fig. 5f, knock-down of DAPK1 significantly inhibited SB203580-regulated anti-inflammation. However, there was difference between oxyHb + DAPK1 KD group and oxyHb + DAPK1 KD + SB203580 in the mRNA expression of *Il-6* and *Il-1β*, which indicated that SB203580-regulated neuroinflammation was not dependent on DAPK1 alone. There was significantly difference between oxyHb group and oxyHb + DAPK1 KD group in the mRNA expression of *Il-1β*, *Il-6* and *Nlrp3.* In conclusion, DAPK1 contributed to P38-regulated LAP and neuroinflammation.

### BECN1 contributed to P38-DAPK1-regulated LAP

**Fig. 6:**
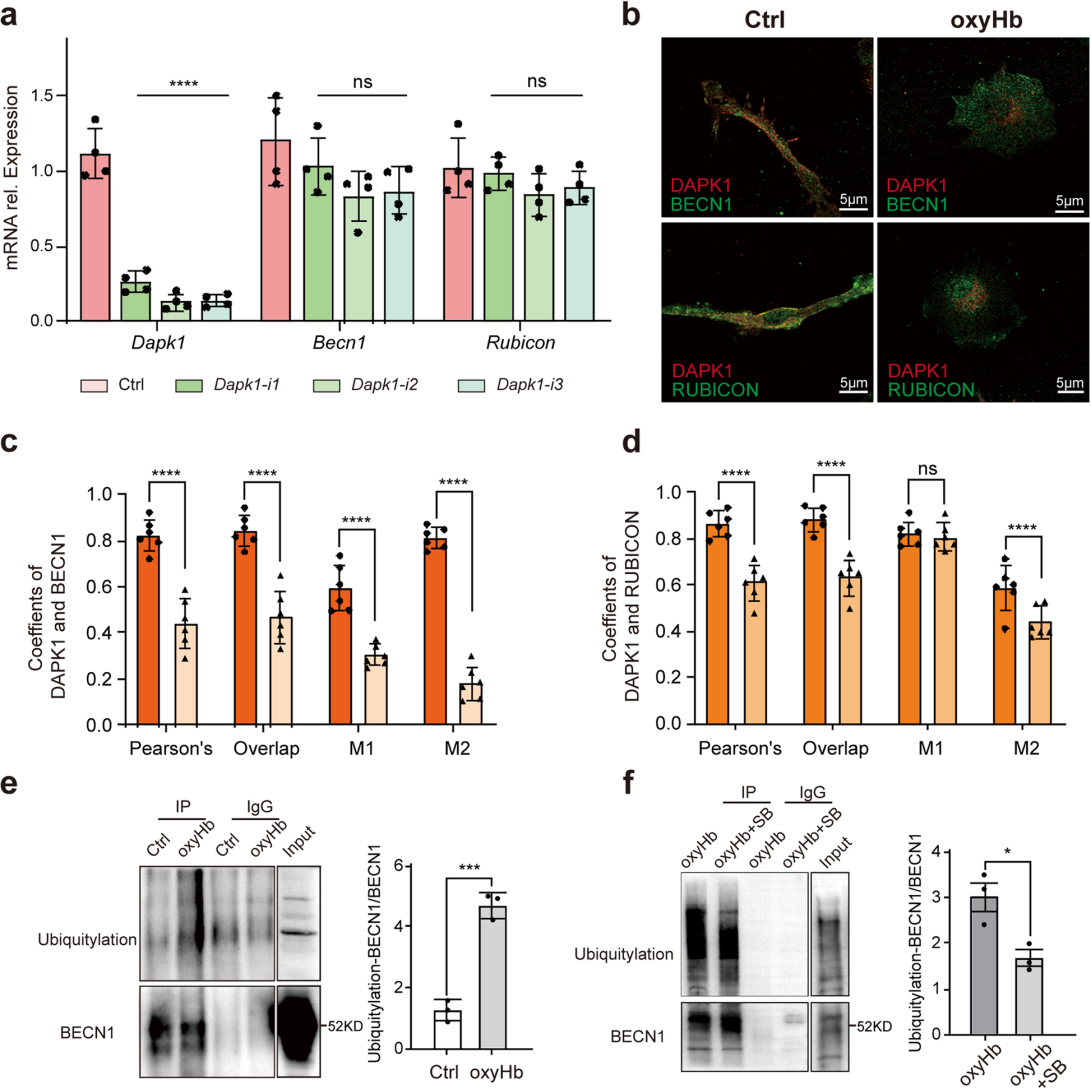
BECN1 contributing to P38-DAPK1-regulated LAP. The mRNA expression of *Dapk1*, *Becn1* and *Rubicon* after transfection of DAPK1-specific plasmid (**a**). Immunofluorescence showing the co-location of DAPK1 and BECN1, DAPK1 and RUBICON (**b**). The change of co-location coefficient between DAPK1 and BECN1 (**c**), DAPK1 and RUBICON (**d**) after oxyHb exposure. Ubiquitylation of BECN1 after oxyHb exposure (**e**) and SB203580 (SB) added (**f**). ****-*P* < 0.0001, ***-*P* < 0.001, *-*P* < 0.05; ns, non-significant. Scale bar: 5 μm

After clarifying the role of P38-DAPK1 axis in the LAP, we wanted to delve into the potential mechanism. We had found that DAPK1 regulated the expression of BECN1, LC3 and RUBICON. However, after knock-down of DAPK1, there was not difference in the mRNA expression of *Becn1* and *Rubicon* (Fig. 6a)*.* Therefore, we speculated that DAPK1 regulated posttranslational modification of BECN1 and RUBICON. With IF, we identified that DAPK1 co-localized with BECN1 and RUBICON as shown in Fig. 6b. With Fiji software, we analyzed the coefficients of colocalization including Pearson’s Coefficients, Overlap Coefficients and Manders' Coefficients in microglia after oxyHb exposure and corrected the coefficients. There was significantly difference in Pearson’s Coefficients, Overlap Coefficients, M1 Coefficients and M2 Coefficients of DAPK1 and BECN1 between control group and oxyHb group (Fig. 6c). However, there was little difference in Manders’ Coefficients of DAPK1 and RUBICON (Fig. 6d). Pearson’s Coefficients and Overlap Coefficients applied to the case that the localization of two different proteins in the cell was related linearly. Manders' Coefficients required only a clean background. M1 represented the ratio of the overlapping portion of the two proteins to DAPK1 and M2 represented the ratio of the overlapping portion of the two proteins to BECN1 or RUBICON. After oxyHb exposure, the localization of DAPK1 and BECN1, RUBICON in microglia was very different. Therefore, the significance of Manders' Coefficients was greater in the study, and BECN1 contributed more to P38-DAPK1-regulated LAP than RUBICON. Considering the difference in protein and mRNA expression of BECN1 induced by P38-DAPK1 axis, we studied the change of ubiquitylation of BECN1 in vitro SAH model. As shown in Fig. 6e, oxyHb exposure significantly induced increased expression of ubiquitylation of BECN1, and SB203580 induced inhibited ubiquitylation of BECN1 limitedly (Fig. 6f). Therefore, Ubiquitylation of BECN1 might affect P38-DAPK1-regulated LAP. In conclusion, BECN1 played an important role in P38-DAPK1-regulated LAP.

## Discussion

In the present research, we studied LAP for the first time in the field of research on subarachnoid hemorrhage. We found the significance of P38-DAPK1 axis in regulating LAP and inflammation of microglia and explored the potential mechanism.

There was increasing concern with microglial phagocytosis considering its importance in improving the prognosis of SAH patients [5, 7]. Different from mainstream studies on microglial phagocytosis, we concentrated on the LAP of microglia to improve the microglial phagocytosis and homeostasis based on “digest me” concept. LAP firstly discovered to be important for the clearance of dead cells was in macrophage [19]. Recently, LAP was identified to facilitate microglia to clear β-Amyloid and mitigate neurodegeneration in Alzheimer’s disease [16]. However, other related studies mainly focused on the immune response [32, 33], and whether LAP contributed to microglial phagocytosis after SAH had not been addressed. In our study, we found that the LAP of microglia was inhibited in all stages in vitro SAH model. We also found that the autophagy and phagocytosis of microglia were inhibited after SAH. Therefore, we took autophagy as a starting point to find the key to regulate LAP. Previous research revealed LAP was different from canonical autophagy although there were many common components [34, 35]. However, we found that the significantly changed signal pathways and genes from canonical autophagy pathways in microglia after SAH were closely associated with LAP, which indicated that autophagy contributed to phagocytosis in microglia. Increasing microglial LAP promoted the phagocytosis of microglia in vitro SAH model. At the same time, we found increasing LAP was accompanied by alleviated inflammation, which indicated the importance of LAP on the homeostasis of microglia.

Many studies have found that MAPK pathway including ERK pathway, JNK pathway and P38 pathway was closely associated with autophagy [21–23]. Among them, P38 pathway was widely popular in the research of autophagy and SAH [36, 37]. However, it was not known whether the MAPK pathway affected LAP. In our study, P38 signal pathway was identified to affect the microglial LAP. OxyHb exposure induced P38 activation, and P38 inhibitor improved microglial phagocytosis and LAP-related proteins including LC3 II, BECN1 and RUBICON. We also confirmed that P38 inhibitor alleviated neuroinflammation. Therefore, P38 activation after SAH in microglia affected the phagocytosis and homeostasis of microglia, and P38 can be an important target for regulating microglial phagocytosis and homeostasis to improve patient prognosis.

DAPK1 was the most significantly changed autophagy-related gene in microglia after SAH in our study. However, there was not concern with DAPK1 in the research of SAH. Previous studies indicated that DAPK1 as a regulator of apoptosis and autophagy was correlated with P38 pathway [24, 31]. In our study, we identified that P38-regulated LAP was dependent on DAPK1. In addition, DAPK1 contributed to P38-regualed neuroinflammation. Oikonomou et al. (2016) had studied the effect of DAPK1 on inflammation and LAP in non-classical fungal autophagy [25]. We confirmed that DAPK1 occupied an important position for the LAP and inflammation in microglia after SAH. It was worth noting that P38-regulated neuroinflammation was partially dependent on DAPK1.

Although Oikonomou et al. (2016) studied the regulation of DAPK1 in LAP and inflammation, they focused their study on how DAPK1 regulated the inflammation but did not explore the mechanism by which DAPK1 regulates LAP [25]. In our study, P38-regulated LAP was dependent on DAPK1, but P38-regulated neuroinflammation was only partially dependent on DAPK1. Therefore, we wanted to delve into the potential mechanism by which the P38-DAPK1 axis regulates the LAP of microglia. After knock-down of DAPK1, the protein expression of BECN1 and RUBICON decreased but the mRNA expression of them was unaffected, which suggested altered protein modification of BECN1 and RUBICON. Colocalization analysis indicated that oxyHb exposure significantly decreased the colocalization coefficient of DAPK1 and BECN1. Previous studies have confirmed that BECN1 plays a core role in regulating autophagy and LAP by affecting multiple cofactors including UVRAG, RUBICON, etc. and downstream molecules including ATG5, ATG7 and so on [38]. Therefore, we thought the LAP regulated by P38-DAPK1 axis was closely associated with BECN1. In recent years, there was increasing concern with ubiquitylation of BECN1 in regulating autophagy [39–41]. In the in vitro SAH model, ubiquitylation of BECN1 increased significantly in microglia and P38 inhibitor inhibited the ubiquitylation of BECN1 to a certain extent. Therefore, ubiquitylation of BECN1 might regulated P38-DAPK1-regulated LAP in microglia after SAH.

In addition, there are still some shortcomings in our study. First, the relationship between LAP and neuroinflammation regulated by the P38-DAPK1 axis has not been explored in depth. It was worthy of further investigation to demonstrate the importance of LAP in the homeostasis of microglia. Second, P38-regulated neuroinflammation does not depend exclusively on DAPK1, and there are other potential mechanisms worth investigating. Finally, the regulation of RUBICON by P38 was not fully consistent with the regulation by DAPK1, suggesting that P38 has a potential regulatory role for RUBICON independent of DAPK1.

## Conclusion

**Fig. 7:**
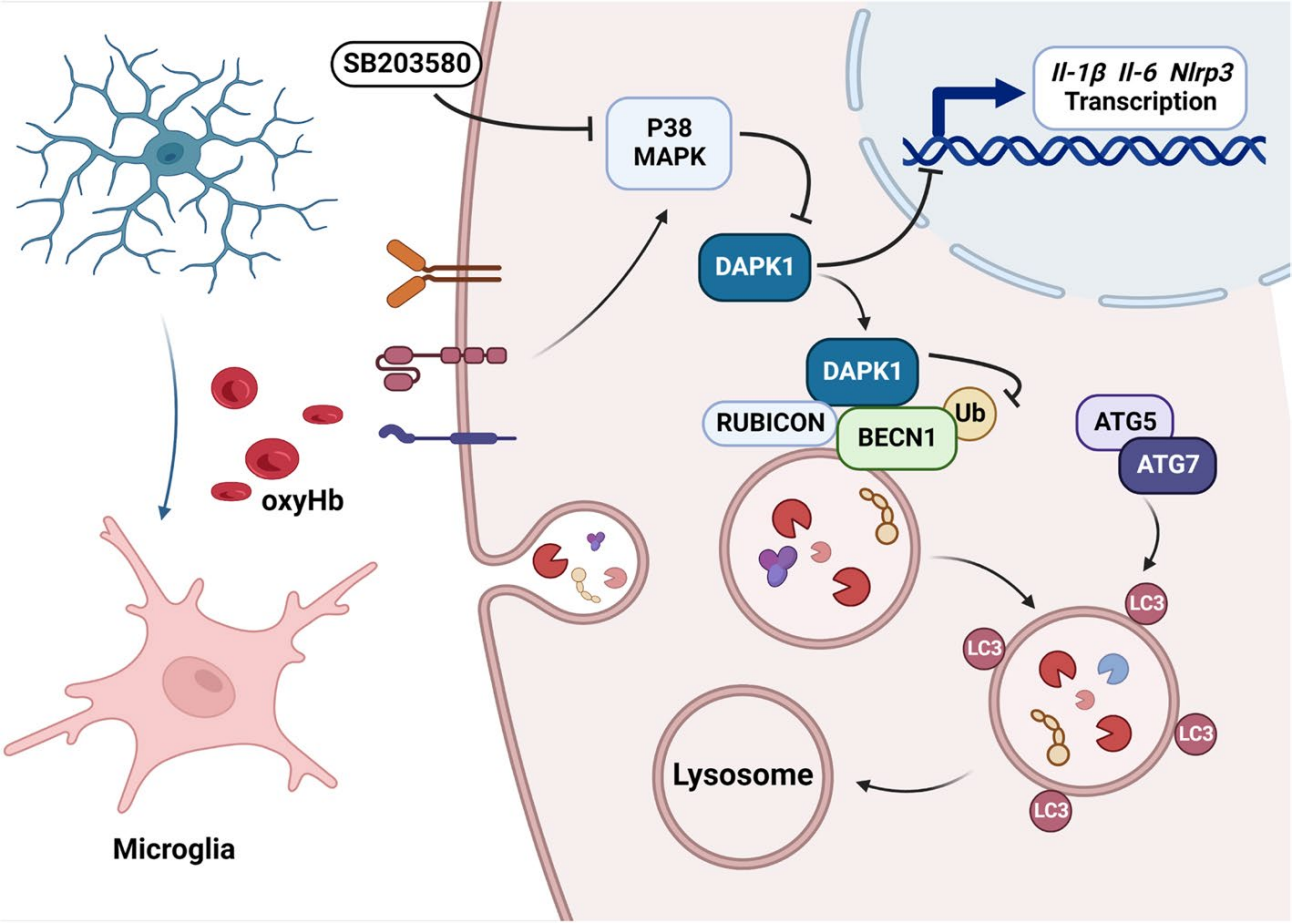
Schematic diagram of our study. OxyHb exposure induced excessive inflammation and impaired phagocytosis. P38 inhibitor (SB203580) increased the expression of DAPK1, which alleviated neuroinflammation and improved LAP by regulating ubiquitylation of BECN1

In conclusion, we have discussed the factors influencing the phagocytosis and homeostasis of microglia from a new perspective in the field of SAH research. Focusing on LAP, we explored a new mechanism of P38-DAPK1 axis regulating the phagocytosis and homeostasis of microglia, reaffirming the central role of BECN1 in autophagy and LAP (Fig. 7).

## Supplementary Information

**Additional file 1.**

**Additional file 2.**

**Additional file 3.**

**Additional file 4.**

## Data Availability

All data generated or analyzed in the study are included in this published article [and its additional files].

## Abbreviations

SAH: Subarachnoid hemorrhage
LC3: Microtubule-associated proteins 1A/1B light chain 3A
LAP: LC3-associated phagocytosis
BECN1: BECLIN-1
MAPK: Mitogen-activated protein kinase
DAPK1: Death associated protein kinase 1
DMEM: Dulbecco's Modified Eagle Medium
FBS: Fetal Bovine Serum
DIV10: 10 Days in vitro
oxyHb: Oxyhemoglobin
WB: Western Blot
qPCR: Quantitative Real-Time Polymerase Chain Reaction
