# Supplementary material for: P38-DAPK1 axis regulated LC3-associated phagocytosis (LAP) of microglia in an in vitro subarachnoid hemorrhage model
Source: Cell Commun Signal. 2023 Jul 21;21:175. doi: 10.1186/s12964-023-01173-6 (PMC10362611; doi:10.1186/s12964-023-01173-6)
Supplement: Supplementary file 2 — Additional file 1. [file 12964_2023_1173_MOESM1_ESM.docx]

| gene | con1 | con2 | con3 | oxyhb1 | oxyhb2 | oxyhb3 |
| --- | --- | --- | --- | --- | --- | --- |
| *Irs4* | 0.1732323 | 0.1218704 | 0.0267114 | 1.9548154 | 2.0703626 | 8.9878617 |
| *Prkcq* | 2.1777011 | 2.3181634 | 2.208063 | 41.531739 | 40.463219 | 65.691568 |
| *Dapk2* | 0.0754837 | 0.0743448 | 0.1629478 | 0.2908534 | 0.5005739 | 0.7449564 |
| *Sqstm1* | 359.09413 | 372.50939 | 357.63379 | 1237.5948 | 1302.982 | 1542.8056 |
| *Rab7b* | 30.956749 | 31.982622 | 30.14115 | 125.73973 | 125.54261 | 96.042845 |
| *Tor3a* | 19.009839 | 16.31858 | 17.192275 | 46.686353 | 49.731751 | 72.358231 |
| *Tor1aip2* | 32.240319 | 31.154731 | 32.482566 | 83.616407 | 80.939066 | 97.656165 |
| *Cflar* | 8.8890147 | 9.1423844 | 9.7669416 | 19.707413 | 20.62143 | 32.37269 |
| *Map2k1* | 47.449342 | 50.307398 | 50.371981 | 116.76972 | 122.7346 | 133.14089 |
| *Prkcd* | 38.518725 | 37.909862 | 41.909061 | 76.699983 | 81.577608 | 113.96038 |
| *Pten* | 108.91196 | 104.98888 | 107.07935 | 72.342411 | 74.836994 | 44.752571 |
| *Prkaa2* | 23.024973 | 21.907524 | 21.624354 | 12.615069 | 14.17833 | 11.51611 |
| *Camkk2* | 31.548621 | 30.468565 | 31.204301 | 15.909803 | 17.461363 | 14.174324 |
| *Atg10* | 13.055605 | 15.805381 | 17.614587 | 7.5109338 | 7.1224082 | 8.5003292 |
| *Pik3r1* | 310.93399 | 295.17693 | 296.9773 | 162.63823 | 162.09783 | 107.6464 |
| *Ulk1* | 153.81601 | 146.38778 | 154.20634 | 72.9175 | 77.472513 | 45.562101 |
| *Ulk2* | 200.19292 | 194.50296 | 197.14961 | 87.711853 | 85.744398 | 73.864622 |
| *Dapk1* | 137.31892 | 132.54034 | 135.08293 | 57.057345 | 61.875893 | 41.841926 |
| *Ddit4* | 255.78468 | 274.30308 | 257.30724 | 101.22627 | 113.09531 | 98.276807 |
| *Mras* | 116.71428 | 115.28192 | 116.57817 | 45.50419 | 44.386672 | 47.727397 |

TPM：Transcripts Per Million
