# Supplementary figures and images for: P38-DAPK1 axis regulated LC3-associated phagocytosis (LAP) of microglia in an in vitro subarachnoid hemorrhage model
Source: Cell Commun Signal. 2023 Jul 21;21:175. doi: 10.1186/s12964-023-01173-6 (PMC10362611; doi:10.1186/s12964-023-01173-6)

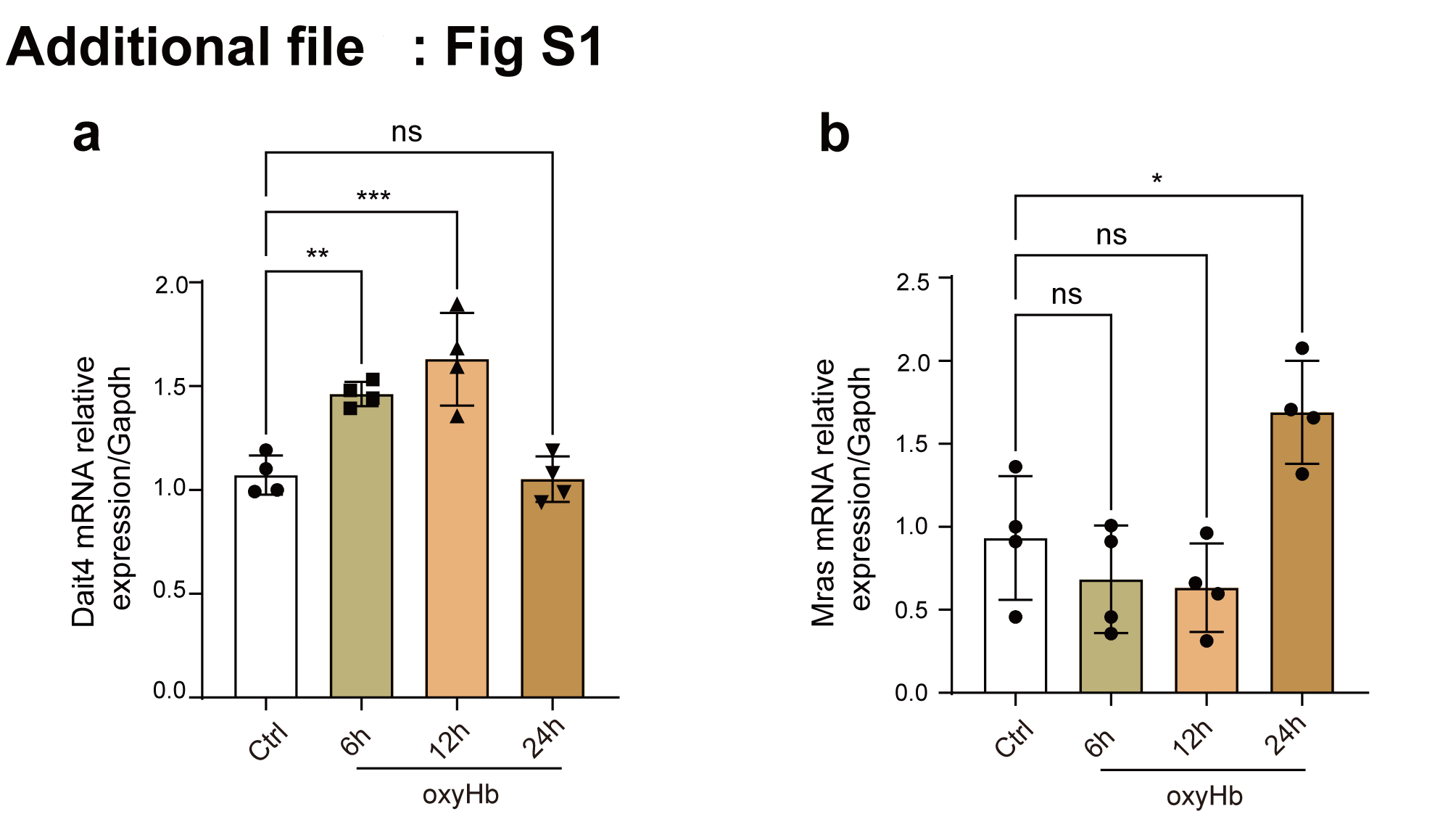

Supplement: Supplementary file 3 — Additional file 2. [file 12964_2023_1173_MOESM2_ESM.tif]

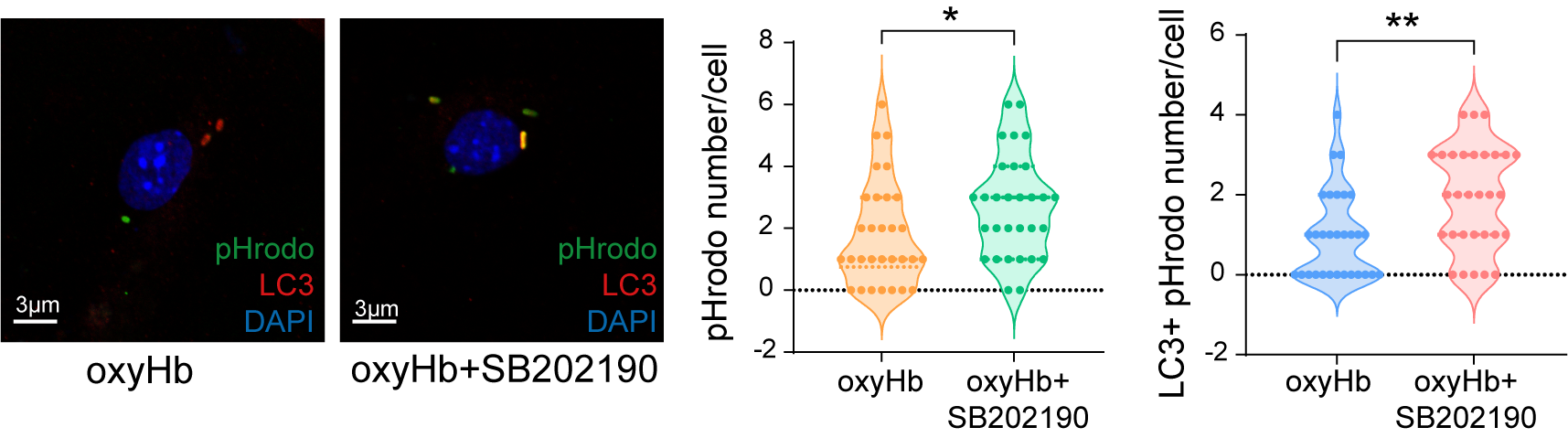

Supplement: Supplementary file 4 — Additional file 3. [file 12964_2023_1173_MOESM3_ESM.tif]

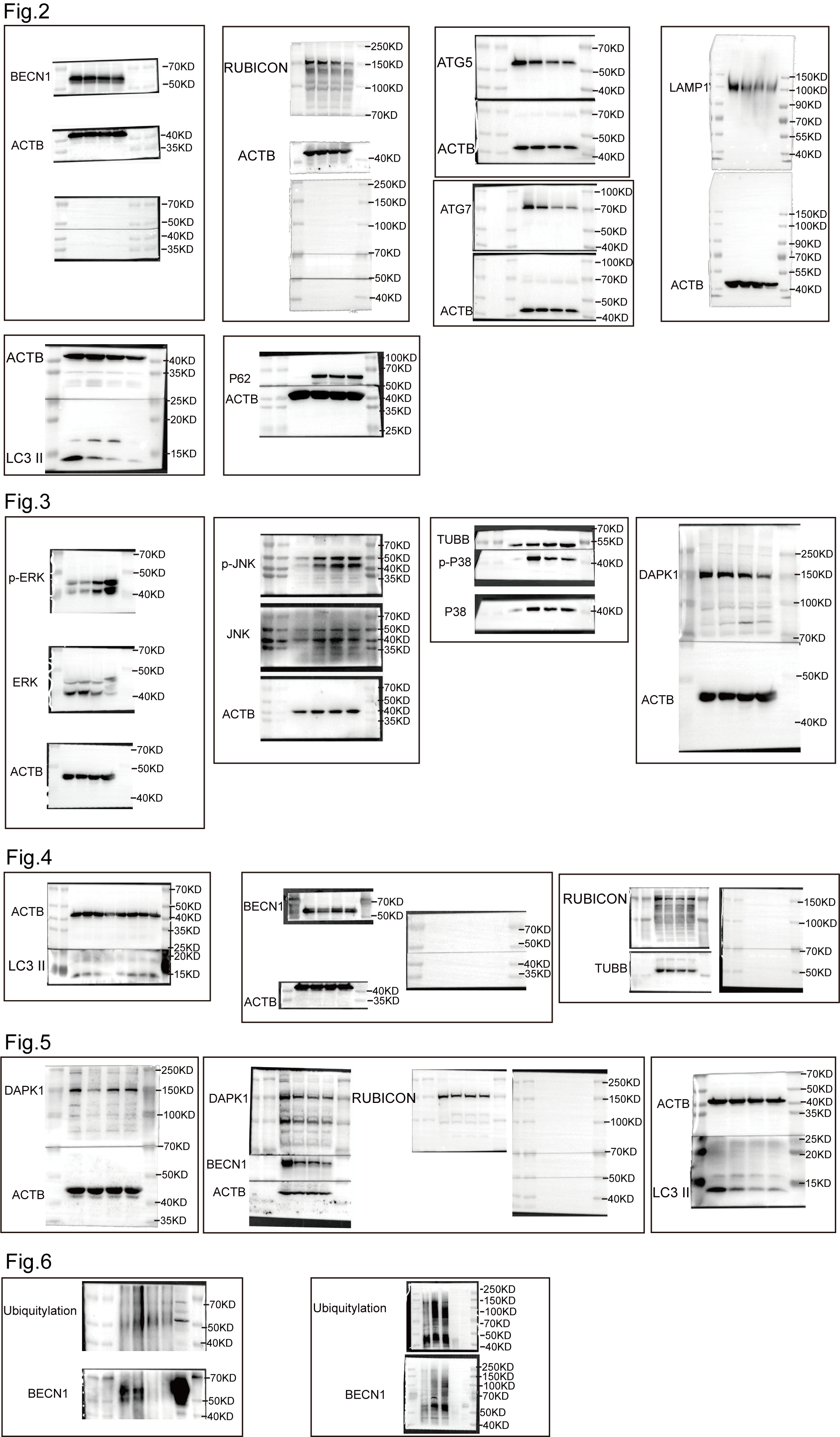

Supplement: Supplementary file 5 — Additional file 4. [file 12964_2023_1173_MOESM4_ESM.tif]
